# Characteristics, Citation Analysis, and Altmetrics Impact of Retracted Papers in Dentistry (2001-2024)

**DOI:** 10.1016/j.identj.2026.109406

**Published:** 2026-02-05

**Authors:** Alireza Jafari, Shohreh SeyyedHosseini, Reza BasirianJahromi

**Affiliations:** a Prosthodontics Department, Bushehr University of Medical Sciences, Bushehr, Iran; b Faculty of Paramedicine, Bushehr University of Medical Sciences, Bushehr, Iran

**Keywords:** Retraction, Dentistry, Publication ethics, Scientific fraud, Social media

## Abstract

**Background:**

Retraction is a mechanism for correcting published scholarly literature and alerting readers to seriously flawed or erroneous content, or ethical issues, in the literature they are reading. The objectives of this study were to identify the reasons for retraction, analyse citations, and describe the scientific and Altmetrics impacts of retracted papers in dentistry, oral health, and medicine.

**Methods:**

The present study was an applied, descriptive-analytical investigation conducted using Scientometric methods and the Altmetrics index. The research population consisted of 231 retracted scientific articles in the subject areas of Dentistry, Oral Surgery, and Medicine, which were indexed in the Web of Science database between 2001 and 2024. Statistical methods, including frequency, mean, and Spearman’s correlation, were employed for data analysis using R software.

**Results:**

The findings showed that out of the 231 retracted articles, 156 articles collectively received 2271 citations. Q1 journals have hosted the most retracted articles. Spain has the highest number of retracted articles in the field of dentistry worldwide. Falsification/fabrication of data is the most important reason for the retraction of articles. Mendeley had the highest share of retracted papers in dentistry among the reference management tools. The correlation coefficient between Altmetrics impact and scientific impact was significant (*P* < .05).

**Conclusion:**

As dentistry and oral health are a pivotal field within the biomedical sciences, they exert a substantial influence on the health of the population. For the preceding decades, it has remained imperative for dentistry researchers to dedicate greater attention to all phases of their research process, encompassing the study design, review process, and publication stage.

## Introduction

Scientific retractions serve as a critical mechanism to uphold the integrity of scholarly literature by addressing errors, misconduct, or irreproducible findings in published research.^1^ In fields such as medicine, dentistry, and oral health, where evidence-based practices directly influence patient care, the dissemination of flawed information can have profound consequences, including misguided treatments and eroded public trust.^2^ The advent of social media platforms has amplified these risks, as retracted papers often continue to garner attention, spreading misinformation rapidly among professionals, patients, and the general public.^3^ This introduction explores the phenomenon of attention to retracted papers in dentistry, oral health, and medicine on social media, integrating a literature review to highlight trends, causes, and implications.

Retractions in medical and dental literature have increased in recent decades, driven by factors such as plagiarism, data fabrication, and duplicate publications.^4^ A systematic review of biomedical retractions identified misconduct as the primary reason, accounting for over 67% of cases, with plagiarism and fraud being prevalent.^5^ In dentistry specifically, a survey of 72 retracted articles revealed redundant publication (20.8%) and plagiarism (18.1%) as leading causes, predominantly in lower-impact journals.^6^ Another comprehensive analysis of 198 retracted dental publications over 23 years echoed these findings, noting plagiarism as the most common reason (38.02%), with India and Spain contributing disproportionately to the total.^7^ Oral health-related retractions, often involving laboratory studies or case reports, further underscore vulnerabilities in these subfields, where ethical lapses such as data manipulation compromise research validity.^8^ During the COVID-19 pandemic, retractions increased, with 143 peer-reviewed articles withdrawn, especially in the health sciences, highlighting how crises exacerbate publication pressures and errors.^1^

Social media’s role in perpetuating attention to these retracted works is particularly concerning. Platforms like Twitter (now X) and Facebook enable rapid sharing, often without context about a paper’s retraction status.^9^ A study analysing Altmetric data found that retracted articles receive 1.2 to 7.4 times more media and social media attention than matched nonretracted ones, even postretraction, due to preretraction buzz.^3^ This ‘attention inertia’ is evident in medicine, where flawed COVID-19 papers continued to attract mentions on social media despite retractions, influencing public discourse and policy.^10^ In dentistry and oral health, similar patterns emerge; for instance, retracted studies on aesthetic procedures or misinformation about treatments like veneers gain traction on platforms, shaping patient behaviours and professional practices.^11^ A scoping review noted that oral health misinformation proliferates on social media, with retracted or debunked claims about tobacco products or dental trends receiving high engagement between 2020 and 2022.^2^ This is compounded by the fact that 89.6% of postretraction citations in dental literature treat the retracted data as reliable, extending to social media shares that amplify unverified content.^12^

The implications for dentistry and oral health are multifaceted. Social media influences patient decisions, with studies showing that exposure to retracted aesthetic dentistry content on platforms leads to dissatisfaction and unethical practices among young dentists.^11^ In medicine broadly, retracted papers on vaccines or treatments, such as those from the COVID-19 era, have fuelled misinformation campaigns on Twitter, delaying retractions from curbing the spread. Altmetric analyses reveal that retracted health articles often receive equivalent or greater social media attention than valid ones, perpetuating harm in areas like oral cancer research or periodontal studies.^10^ For example, a retracted paper on digital dentistry tools continued to be discussed online, ignoring ethical concerns.^3^ This dynamic is worse in oral health, where viral trends debunked in retracted literature – such as unproven whitening methods – persist on TikTok and Instagram, influencing public perceptions and adherence to evidence-based care.

Efforts to mitigate this include better retraction visibility, such as watermarks on PDFs, yet 34% of retracted dental articles lack them, allowing unchecked sharing.^7^ Journals must enforce COPE guidelines more rigorously, as delays in retraction notices – averaging 17 months in dentistry – exacerbate social media dissemination.^13^ In medicine, cross-platform tracking shows that retracted papers receive sustained attention on 14 platforms, including social media, underscoring the need for digital interventions.^9^ For oral health professionals, this calls for education on e-professionalism, as social media use can inadvertently promote retracted content.^14^

While retractions aim to correct the record, social media often sustains attention to flawed papers in dentistry, oral health, and medicine, risking misinformation and patient harm. Based on the above statements, the current research aims to investigate the reasons for the retraction of articles in the field of dentistry, as well as the extent to which they receive attention on various social media platforms. Our hypothesis is based upon the evidence that retracted articles with higher Altmetric attention also tend to have higher traditional scientific impact.^3^ Thus, the study hypothesis is manifested as follows:

There is a significant relationship between Altmetrics impact and Scientific impact (citations) of retracted articles in dentistry.

## Methods and materials

The present research was an applied, descriptive-analytical study that was conducted using Scientometric methods and utilizing the Altmetrics index. The research population consisted of scientific articles in the subject areas of Dentistry, Oral Surgery, and Medicine, which were indexed in the Web of Science (WoS) database. This database is highly regarded by researchers in the field. The data for this study were retrieved from the WoS database on 24 July 2025. To execute this process, the ‘Dentistry, Oral Surgery & Medicine’ field was selected in the first search box under the ‘Web of Science Categories’ section. In the second search box, the ‘Document Type’ field was selected with the options ‘Retraction’ and ‘Retracted Publication’. This search strategy yielded 253 retracted articles from researchers worldwide in the fields of Dentistry, Oral Surgery, and Medicine. Following deduplication, 12 duplicate records were removed from the results. The remaining 241 retracted articles were retrieved and analysed individually using the ‘Altmetric It’ plugin, which had previously been added to the browser’s bookmarks. If the article had an Altmetrics score, it would be displayed, and clicking on the score would show further details. A total of 241 retracted articles from this field were retrieved from the WoS, with seven articles lacking a DOI or PMID, thus resulting in the absence of an Altmetrics score for these articles. Consequently, these articles were excluded from the study, resulting in a final sample of 231 retracted articles.

The Altmetrics Bookmarklet tool was selected due to its credibility, significance, and widespread use among researchers. The utilization of this instrument entailed various metrics, including the number of downloads, the level of attention accorded to the articles, the frequency of mentions on social media platforms, news outlets, blogs, and across a range of Web 2.0-based services. The Altmetrics Bookmarklet is an application that collects data related to journal articles from news, blog posts, tweets, and research article-related posts. The tool in question assigns a score based on the information provided by each data source. Accordingly, the Altmetrics Bookmarklet tool was utilized to comprehensively analyse all mentions of the retracted articles in Dentistry, Oral Surgery, and Medicine, videos, texts, and writings. This analysis was conducted without the imposition of time limitations, and various scores for these articles were obtained. The aggregation of these scores was designated as an Altmetrics score, which is indicative of the degree of sharing, attention, and utilization of these articles on social media. The Altmetrics score assigned is indicative of the quantity and quality of the attention a document has received on social media. The attention scores for each article were retrieved from the Altmetric tool, available at https://www.altmetric.com. The tool was used on 24 July 2025. Given that Altmetrics scores are subject to regular updates each time an article is shared, the scores collected for analysis are representative only of the specific point in time at which they are recorded. However, it should be noted that data collection for Altmetrics in this study was conducted after all articles had been retrieved.

Citation data for articles in this field were extracted from the WoS citation report section. To retrieve the status of journals containing retracted articles in the field of *Dentistry, Oral Surgery, and Medicine*, each journal was searched in the Journal Citation Reports, and relevant information was retrieved. To retrieve the reasons for the retraction of articles in this field, each article was searched in the Retraction Watch Database at https://retractiondatabase.org/RetractionSearch.aspx, and the relevant information was retrieved. Finally, the obtained data were analysed using R software. To measure the correlation between scientific impact and Altmetric impact, the Kolmogorov-Smirnov test was initially conducted. Since the data were not normally distributed, the Spearman correlation test was used.

## Findings

### Analysing the status of Altmetrics impact and the scientific impact of rejected articles in the field of Dentistry, Oral Surgery, and Medicine by year

The results of the examination of attention to rejected articles in the field of Dentistry, Oral Surgery, and Medicine show that out of a total of 231 retracted articles, 94 articles (40.69%) were mentioned on social media. In comparison, 137 articles (59.31%) were not referenced on any social media platforms.

According to Table 1, among the 231 retracted articles in the field of Dentistry, Oral Surgery, and Medicine, the highest number of retracted articles was in the year 2018 (29 articles), while the lowest was in the years 2001, 2002, and 2006 (1 article). Additionally, the highest number of retracted articles with citations is from 2012 (16 articles), and the lowest number of cited articles is from 2001, 2002, and 2006 (1 article each). Furthermore, the highest number of retracted articles with Altmetric scores is from the year 2018 (16 articles), while the lowest number of retracted articles with Altmetric scores is from the years 2004 to 2007 and 2025 (0 articles). The highest average citations for retracted articles corresponds to the year 2004 (75.3 citations), and the lowest is for the year 2024 (0.2 citations). The highest average Altmetric score for retracted articles is also from the year 2009 (5.7 scores), while the lowest is from the years 2004 to 2007 (0 scores).

**Table 1:** Status of retracted articles in Dentistry, Oral Surgery, and Medicine by time period.

Y	Number of retracted articles	Number of retracted articles with citations	Total citations	Average of citations	Number of retracted articles with an Altmetrics score	Total Altmetrics score	Average Altmetrics score of retracted articles
2001	1	1	44	44	1	1	1
2002	1	1	2	2	1	1	1
2003	3	2	69	23	1	3	1
2004	3	3	226	75.3	0	0	0
2005	2	2	121	60.5	0	0	0
2006	1	1	8	8	0	0	0
2007	3	3	27	9	0	0	0
2008	6	5	154	25.7	3	13	2.2
2009	3	3	47	15.7	2	17	5.7
2010	4	4	107	26.8	2	9	2.3
2011	7	7	133	19	5	24	3.4
2012	17	16	192	11.3	7	32	1.9
2013	8	6	165	20.6	1	5	0.6
2014	15	13	293	19.5	9	43	2.9
2015	15	14	181	12.1	9	35	2.3
2016	15	12	69	4.6	9	24	1.6
2017	10	9	109	10.9	8	27	2.7
2018	29	14	103	3.6	16	51	1.8
2019	13	9	105	8.1	4	16	1.2
2020	15	5	27	1.8	2	7	0.5
2021	17	10	38	2.2	4	6	0.4
2022	10	5	18	1.8	3	30	3
2023	12	7	29	2.4	3	19	1.6
2024	21	4	4	0.2	4	47	2.2
2001-2024	231	156	2271	9.7	94	410	1.8

Moreover, according to the statistics provided in Table 1, out of the 231 retracted articles, 156 articles collectively received 2271 citations. Among these retracted articles, 94 were shared on social media, achieving a total Altmetrics score of 410.

### Status of countries with retracted articles in the field of Dentistry, Oral Surgery, and Medicine

To outline the status of countries with retracted articles in the field of Dentistry, Oral Surgery, and Medicine, the affiliation of the corresponding author for each article was taken into consideration (Table 2).

**Table 2:** Countries of the corresponding author in retracted articles of Dentistry, Oral Surgery, and Medicine.

Country	Retracted articles	Country	Retracted articles
Spain	61	Sweden	2
India	27	New Zealand	2
China	23	Nepal	2
Japan	19	Egypt	2
USA	16	Netherlands	1
Brazil	10	Switzerland	1
UK	8	Singapore	1
Italy	8	Kuwait	1
Germany	7	Malaysia	1
Iran	6	Mexico	1
Taiwan	4	Sri Lanka	1
Norway	4	Jordan	1
Thailand	3	Ireland	1
Saudi Arabia	3	Israel	1
Greece	3	Indonesia	1
Canada	3	Chile	1
Turkey	2	Amman	1
Tunisia	2	Australia	1

According to Tables 2 and 3, Spain has the highest number of retracted articles in the field of Dentistry, Oral Surgery, and Medicine, with 61 articles (26.40%). Following Spain, India ranks second with 27 articles, accounting for 11.68% of the retracted articles; China is third with 23 articles (9.95%), and Japan ranks fourth with 19 articles (8.22%) of the retracted articles.

**Table 3:** Reasons for retraction in Dentistry, Oral Surgery, and Medicine papers.

Reasons for retraction	Retracted publication
Falsification/fabrication of data	73
Investigation by journal/publisher	69
Unreliable results and/or conclusions	67
Plagiarism of an article	58
Concerns/issues about data	55
Duplication of/in article	53
Error in data	42
Ethical violations by the author	41
Objections by author	35
Lack of IRB/IACUC approval and/or compliance	24

### Reasons for the retraction of articles in the field of Dentistry, Oral Surgery, and Medicine

Articles are retracted for various reasons. Table 4 presents the primary reasons articles in the fields of Dentistry, Oral Surgery, and Medicine have been retracted.

**Table 4:** Correlation between Altmetrics impact and scientific impact (citations).

Indices	*N*	*R*	*P*
Altmetrics impact scientific impact	231	0.21	.001

According to Table 3, among the reasons for retraction of articles in the field of Dentistry, Oral Surgery, and Medicine, the most frequent cause is falsification/fabrication of data, which accounts for 73 rejected articles. Following that, in order, are the reasons: Investigation by Journal/Publisher for 69 articles, Unreliable Results and Conclusions for 67 articles, and Plagiarism of Article for 58 articles. A single article may be simultaneously retracted for multiple reasons based on the available data from the Retraction Watch Database. Therefore, the total number of reasons for retraction exceeds the total number of articles (231 cases).

### Social networks publishing retracted articles by researchers in Dentistry, Oral Surgery, and Medicine

**Fig. 1:**
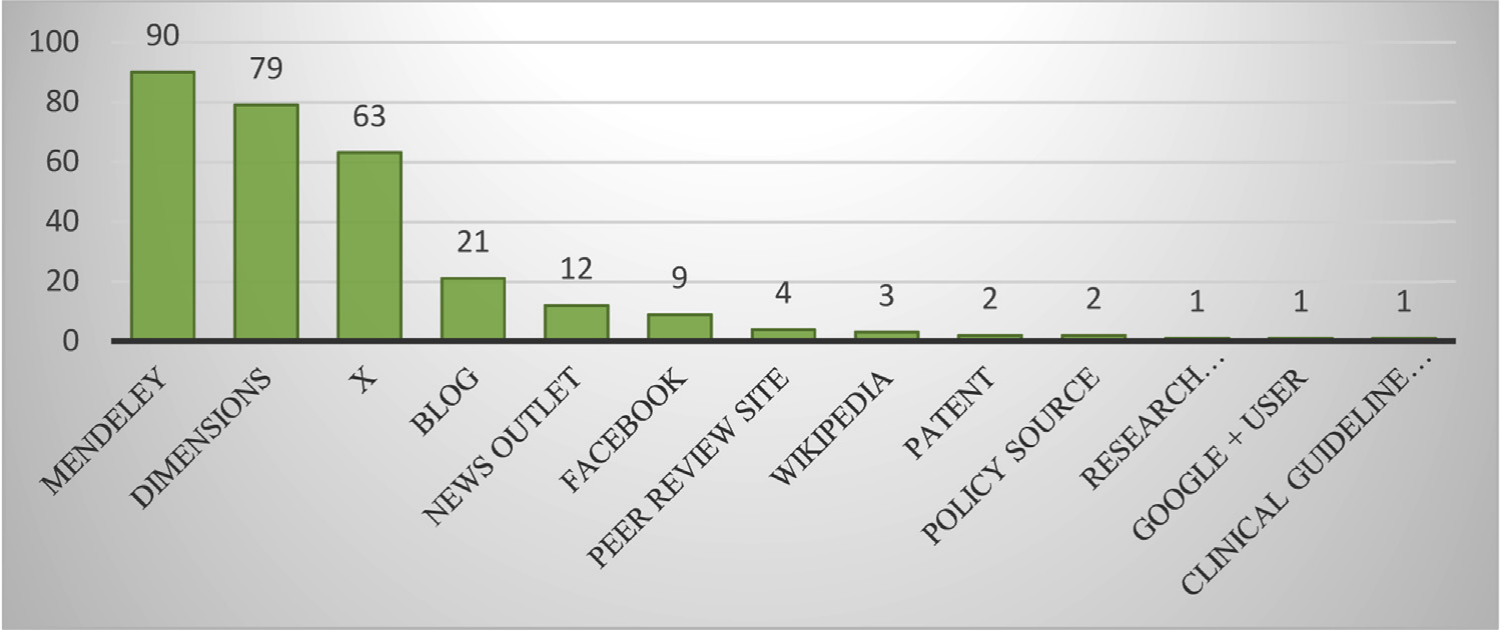
Distribution of social networks sharing retracted articles with Altmetric scores by researchers in the field of Dentistry, Oral Surgery, and Medicine.

According to Figure 1, in the analysis of social networks referencing retracted articles in the field of Dentistry, Oral Surgery, and Medicine, Mendeley had the highest share among the reference management tools; 90 articles (95.74%) from researchers in the field of Dentistry, Oral Surgery, and Medicine were cited 4596 times in Mendeley.

Following Mendeley, the Dimension social network had 2nd place, with 79 retracted articles from researchers in the field (84.04%) shared 1466 times. After Dimension, X had 63 rejected articles (67.02%) shared 113 times by 97 individuals, encompassing a total of 1945,204 Flowers. Next, Blog had 21 articles (22.34%) shared 24 times by 22 individuals, News Outlet had 12 articles (12.77%) shared 22 times, Facebook had 9 articles (9.57%) shared 9 times, Peer Review site had 4 articles (4.26%) shared 4 times, Wikipedia had 3 articles (3.19%) shared 3 times, while Patent and Policy source each had 2 articles (2.13%) shared 2 times. Research Highlight Platform, Google Plus, and Clinical guideline source each had 1 article (1.06%) shared 1 time, making them other significant social networks that referenced retracted articles in the field of Dentistry, Oral Surgery, and Medicine.

### Distribution of retracted articles by researchers in the field of Dentistry, Oral Surgery, and Medicine on X based on the countries of the submitters and the roles of the submitters in the community

The geographical distribution of posts in the Altmetrics explorer database, based on the information available in the profiles of the posters and the geographical tags of the posts, was as follows: the United States had 18 posts, the United Kingdom and Spain each had 6 posts, Canada had 3 posts, Mexico had 2 posts, and other countries each had one post. The United States accounted for the highest percentage of posts for these articles. Due to incomplete information in the profiles of the posters on X, it was not possible to identify their geographical locations for 55 posts of the retracted articles in the field of Dentistry, Oral Surgery, and Medicine.

**Fig. 2:**
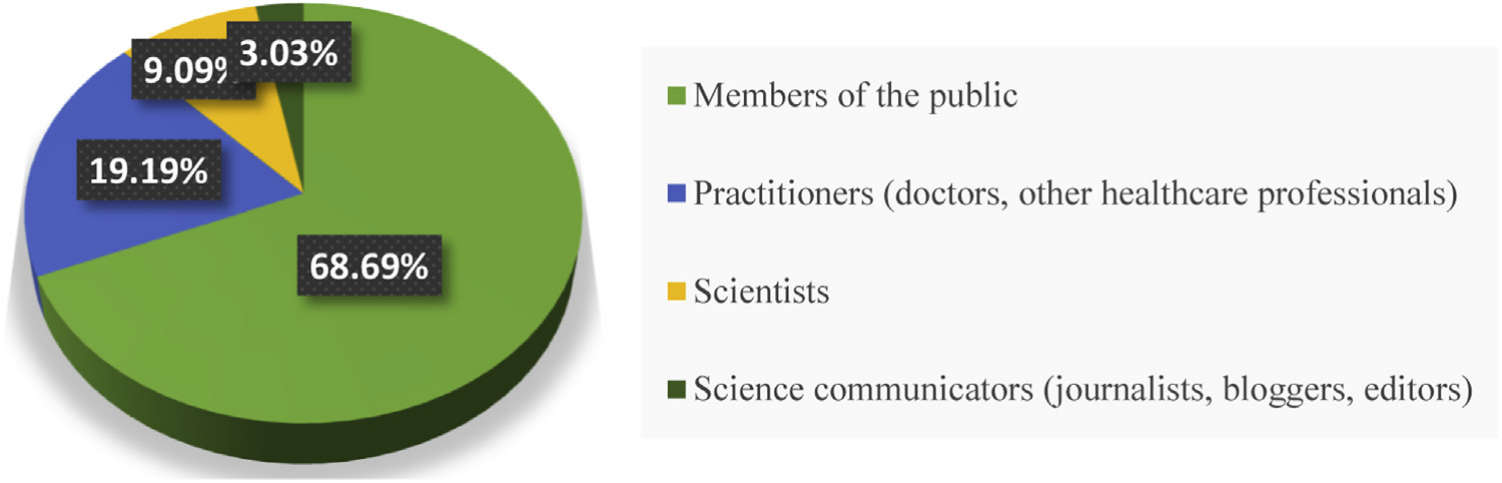
Distribution of posts of retracted articles in the Dentistry, Oral Surgery, and Medicine field in X, by the roles of posters in the community.

The findings in Figure 2 indicate that a total of 68.69% of all posts debunking articles in the field of Dentistry, Oral Surgery, and Medicine on X are made by Members of the public (nonexperts). Thus, laypeople (nonexperts) represent the largest share of posts regarding debunked articles in the field of Dentistry, Oral Surgery, and Medicine on this social network. Following them are Practitioners (doctors, other healthcare professionals) at 19.19%, Scientists at 9.09%, and Science communicators (journalists, bloggers, editors) at 3.03%, ranking next in terms of the volume of posts related to debunked articles in this field on the social network.

### Distribution of citations for retracted articles in the field of Dentistry, Oral Surgery, and Medicine in Mendeley according to the roles of the citing authors in the community

**Fig. 3:**
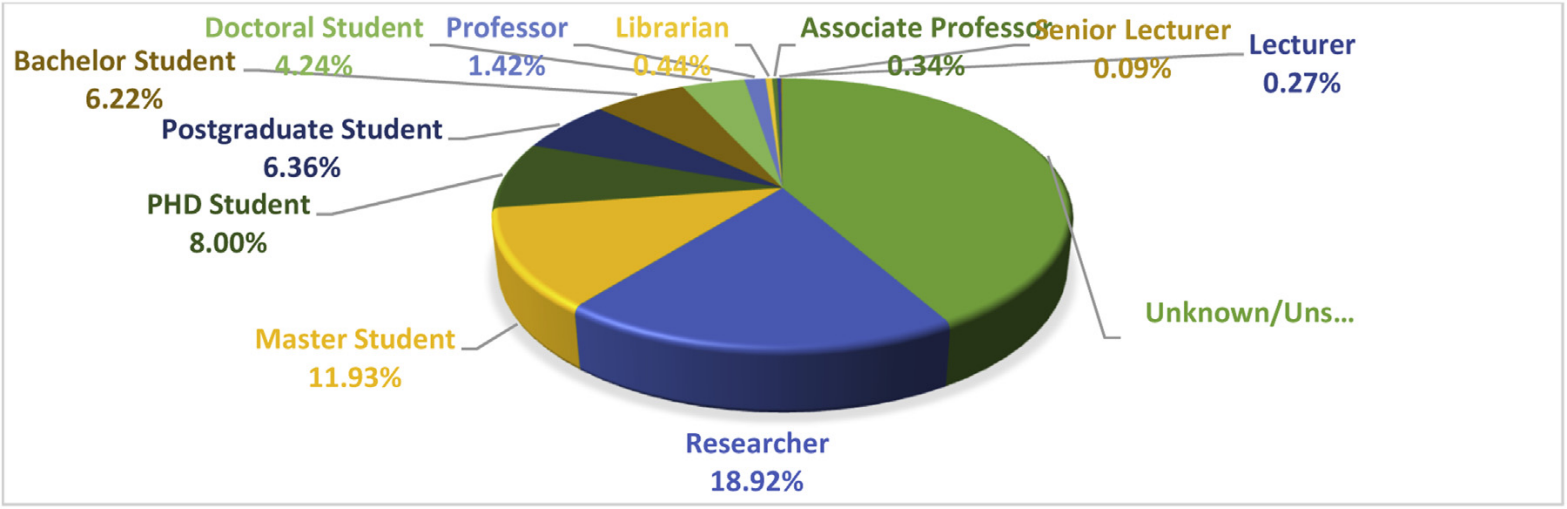
Distribution of citations for retracted articles in the field of Dentistry, Oral Surgery, and Medicine in Mendeley according to the roles of the citing authors in the community.

The findings in Figure 3 show that a total of 18.92% of all references for retracted articles in the field of Dentistry, Oral Surgery, and Medicine on Mendeley are by Researchers.

Researchers hold the largest share of references to retracted articles in this field on Mendeley. Following them are master students with 11.93% and PhD Students with 8.00%, ranking next in terms of the number of references to retracted articles in the field of Dentistry, Oral Surgery, and Medicine on Mendeley. Additionally, in 41.78% of Mendeley references, it was not possible to determine the roles of the referees due to incomplete profile information of the submitters.

### Distribution of references to retracted articles in the field of Dentistry, Oral Surgery, and Medicine in Mendeley, categorized by subjects

**Fig. 4:**
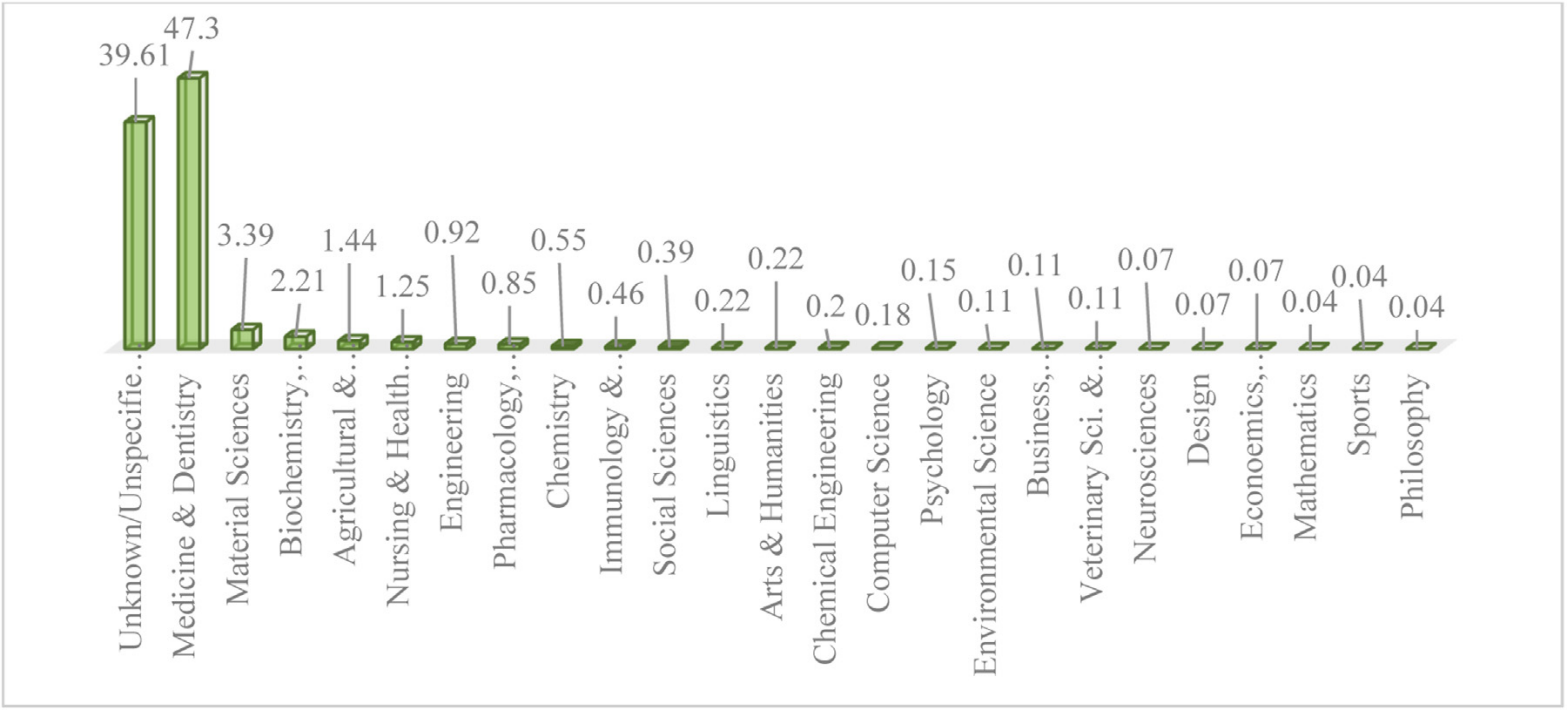
Distribution of references to retracted articles in the field of Dentistry, Oral Surgery, and Medicine in Mendeley, categorized by subjects.

The findings of Figure 4 indicate that a total of 47.30% of the subject area of articles citing retracted articles in the field of Dentistry, Oral Surgery, and Medicine in Mendeley are related to the subject area of Medicine and Dentistry.

Researchers and individuals working in this subject area have the largest share of citations to retracted articles in the field of Dentistry, Oral Surgery, and Medicine in Mendeley. Following this, the subject areas of Material Sciences with 3.39% and Biochemistry, Genetics, and Molecular Biology with 2.21% are in the next ranks in terms of citations to retracted articles in the field of Dentistry, Oral Surgery, and Medicine in Mendeley. Furthermore, in 39.61% of Mendeley citations, it was not possible to identify the subject areas of the citing authors due to incomplete profile information of the submitters.

### The correlation between Altmetrics impact and the scientific impact (citations) of retracted articles in the field of Dentistry, Oral Surgery, and Medicine

To test study hypothesis, we utilized the correlation test between Altmetrics impact and Scientific impact (number of citations). So, the Kolmogorov–Smirnov test was initially performed. The calculated z-value in the Kolmogorov–Smirnov test for Altmetrics impact and Scientific impact was not significant (*P* > .05). Therefore, Altmetrics impact and scientific impact do not have a normal distribution, and nonparametric analyses (Spearman correlation coefficient) should be used to measure the correlation between these two variables.

As shown in Table 4, the correlation coefficient between Altmetrics impact and Scientific impact was significant (*P* < .05).

## Discussion

This study aimed to comprehensively investigate and report the characteristics of retracted articles in the field of Dentistry, Oral Surgery, and Medicine. A total of 231 retracted articles from the Dentistry, Oral Surgery, and Medicine category of the WoS database were retrieved after removing duplicates. The WoS database makes it clear when an article has been retracted by including the term ‘retracted’ right in the title. This transparent labelling helps researchers quickly identify and navigate around articles that are no longer considered credible. The findings of this section indicate that the first retraction of articles in this field dates back to 2001, and the number of retracted articles has been increasing until 2024, with fluctuations in some years. These findings align with other studies conducted in the field of dentistry^15^^,^^16^ and other subject areas.^17^, ^18^, ^19^, ^20^, ^21^, ^22^

The average citation impact and Altmetrics impact also fluctuated significantly in different time periods. Among these, the highest average citation rate for retracted articles was in 2004, and the lowest was in 2024. Similarly, the highest average Altmetrics score for retracted articles was in 2009, and the minimum was between 2004 and 2007.

According to information from Altmetric, a review of retracted articles in Dentistry, Oral Surgery, and Medicine published in the WoS database indicates that less than half of the articles (40%) out of 231 articles have received attention on social media. Perhaps one reason for this is the retraction of these articles; however, it is natural to say that a definitive statement on this matter requires further studies.

The present study’s results indicated that Spain, followed by India, had a higher number of retracted articles in the field of Dentistry, Oral Surgery, and Medicine compared to other countries; a previous study in the field of dentistry had also reached this conclusion.^23^ However, other studies in the field of dentistry have shown that India has the highest number of retracted articles.^15^^,^^16^^,^^24^, ^25^, ^26^ The previous evidence indicates that more dental retractions are *recorded* from India, Japan, and Spain, likely due to a combination of volume of output, systemic incentives, and weaknesses in research governance.^27^ Available studies emphasize that these are structural and policy issues rather than simple individual failings.

Furthermore, the results of the present study showed that the most common reasons for article retraction in the field of Dentistry, Oral Surgery, and Medicine were Falsification/Fabrication of Data, Investigation by Journal/Publisher, and Unreliable Results and/or Conclusions. While some previous studies have identified misconduct – manipulation, fabrication, and falsification of data – as the most common reason for the retraction of retracted articles.^15^^,^^16^^,^^21^^,^^25^^,^^26^^,^^28^ Previous studies have identified plagiarism as the most common reason for article retraction.^17^, ^18^, ^19^^,^^23^ In addition, some studies have identified the publication of duplicate material as the most common reason for article retraction.^24^^,^^29^ Fabrication of data can be considered one of the common factors in which authors play a key role in its formation. Authors may fabricate data for several reasons, often stemming from pressures in the academic and research environment. Some motivations may include pressure to publish, desire for recognition and career advancement, competitive environment, and fear of failure.^30^ Faced with the crisis of data fabrication and falsification by authors, journals, and publishers have not been idle. The findings of the current research indicate that journals and publishers in the field of Dentistry, Oral Surgery, and Medicine, through careful examination of articles suspected of research misconduct, have themselves identified a significant portion of these research violations.

A review of various social media networks indicates that networks such as Mendeley reference management tool, Dimensions, X, News Atlet, Facebook, blogs, patents, Wikipedia, Policy Source, Research Highlight Platform, Google Plus, Peer Review, and Clinical guideline are tools for sharing retracted articles in the field of Dentistry, Oral Surgery, and Medicine. Among these, the Mendeley reference management tool, Dimensions, and X (formerly Twitter) have the highest statistics and are more popular than other social media. The importance of these three media in sharing scientific articles has also been mentioned in previous studies.^31^, ^32^, ^33^, ^34^, ^35^, ^36^, ^37^, ^38^ The reason for this can most likely be attributed to the greater reputation and higher acceptance of these three social networks among the audience. It should be noted that Mendeley, Dimensions, and X (Twitter) show many retracted articles mainly because they are large, research-focused platforms whose metrics are directly harvested by Altmetric services, not because they uniquely ‘cause’ retractions. In practice, they serve as major repositories and dissemination channels for all papers, so retracted papers are highly visible there as a by-product of their overall coverage.^3^ Furthermore, Mendeley is a reference manager and academic social network whose users store copies or records of published papers; a classic study found that personal Mendeley libraries contained records for about 75% of a sample of retracted articles, even though almost none of these records were flagged as retracted in the interface. This high coverage of the literature (including retracted work) makes Mendeley appear to hold many retracted papers.^39^

An analysis of the posts of retracted articles in Dentistry, Oral Surgery, and Medicine on X showed that the United States, Spain, and England had a greater share of these articles. The high share from Spain can be attributed to the high rate of retracted articles from this country and, consequently, the sharing of articles by the lead authors. Previous studies acknowledged the greater contribution of the United States and England in scientific article posts on X.^40^^,^^41^

Dentistry, oral surgery, and medicine. The findings also indicated that ordinary individuals hold the largest share of retracted articles in this field on X. One possible reason for such a high share could be the significant presence of ordinary users with various levels of education in their X profiles. Another reason may be the interest of ordinary individuals in following research related to Dentistry, Oral Surgery, and Medicine. Dental professionals and practitioners of this profession had the next highest share after ordinary individuals. The findings also revealed that researchers and, following them, students (both master’s and PhD) had the highest share in citing retracted articles in Mendeley. This result may reflect the interest of students – along with the researchers themselves – in studying and sharing articles. Other research has also shown that students have a significant share in referencing articles in the Mendeley reference management tool.^42^^,^^43^

A thematic review of retracted articles in the fields of Dentistry, Oral Surgery, and Medicine with the highest citation rates in Mendeley revealed that medicine and dentistry had the most references to these retracted research outputs in Mendeley. Previous researchers have identified medicine and dentistry as fields with the most presence on social media.^30^^,^^34^^,^^39^^,^^44^, ^45^, ^46^, ^47^. This may be related to the short half-life of articles in this domain. Moreover, since researchers in the fields of medicine and dentistry continually seek new treatment methods, they have a greater interest in publishing their research through social media.^42^ This is particularly true for the current study, which focuses on Dentistry, Oral Surgery, and Medicine, areas that typically receive more citations in the medical and dental fields.

Additionally, this study’s findings indicated a significant correlation between the scientific influence of retracted articles in Dentistry, Oral Surgery, and Medicine and their Altmetric impact. Previous studies have also reported a significant statistical relationship between the scientific influence of retracted articles and their Altmetric impact.^21^

## Conclusion

The primary objective of retraction is to rectify inaccuracies in the scientific literature and uphold the integrity of the scientific community. Dentistry and oral health are a pivotal field within the biomedical sciences, exerting a substantial influence on the health of the population. This study offered a comprehensive overview of retracted publications in the field of Dentistry, Oral Surgery, and Medicine, emphasizing their bibliometric and Altmetric characteristics. The analysis of 231 retracted articles indexed in the WoS revealed an increasing trend in retractions from 2001 to 2024, with fluctuations across years – an observation consistent with previous research in both dental and broader scientific disciplines. Variations in citation and Altmetric impact across time further highlighted that retracted articles remain visible and influential even after withdrawal, suggesting that scholarly and public attention often persists beyond retraction events.

Finally, the study faced several limitations, including variability in the content of retraction notices because journals and publishers used different policies for reporting them; in some cases, access to the notices was also limited. The research data were collected exclusively from the WoS categories of Dentistry, Oral Surgery, and Medicine. Because the data came from a single database, we may have overlooked some relevant articles in this field and were unable to include them in the analysis.

## Funding

There is nothing to declare.

## Author contributions

All authors listed have made a substantial, direct, and intellectual contribution to the work and approved it for publication. Shohreh SeyyedHosseini and Reza BasirianJahromi are responsible for the first draft of the manuscript. Shohreh SeyyedHosseini and Alireza Jafari are accountable for data collection and analysis. Alireza Jafari, Reza BasirianJahromi, and Shohreh SeyyedHosseini guided and finalized the study.

## Conflict of interest

The authors declare that they have no known competing financial interests or personal relationships that could have appeared to influence the work reported in this paper.

## Footnotes

1 https://retractionwatch.com/retracted-coronavirus-covid-19-papers/

2 https://www.cda.org/newsroom/patient-care/oral-health-misinformation-is-rampant-on-social-media-cda-journal-investigates/

3 https://www.intechopen.com/journals/4/articles/428
